# SIRT1 Is the Target Gene for 2,3,5,4’-Tetrahydroxystilbene-2-O-β-D-Glucoside Alleviating the HUVEC Senescence

**DOI:** 10.3389/fphar.2020.542902

**Published:** 2020-09-08

**Authors:** Yan Guo, Wenxue Fan, Yuefeng Xie, Shuyu Cao, Haitong Wan, Bo Jin

**Affiliations:** ^1^ College of Life Science, Zhejiang Chinese Medical University, Hangzhou, China; ^2^ College of Basic Medicine and Public Health, Zhejiang Chinese Medical University, Hangzhou, China

**Keywords:** human umbilical vein cells, senescence, SIRT1, 2,3,5,4’-tetrahydroxystilbene-2-O-β-d-glucoside, hydrogen peroxide

## Abstract

This study aimed to explore the effects of 2,3,5,4’-tetrahydroxy-stilbene-2-O-β-d-glucoside (TSG) on the senescence of human umbilical vein cells (HUVEC) induced by hydrogen peroxide (H_2_O_2_) and to identify the potential targets mediating its protective action. HUVEC cells pre-treated with TSG for 24 h were exposed to H_2_O_2_ treatment. TSG significantly decreased H_2_O_2_-induced cellular senescence, as indicated by reduced senescence-associated β-galactosidase (SA-β-gal) positive staining, the proportion of cells in the G1 phase, cell apoptosis, p21, and plasminogen activator inhibitor-1 (PAI-1) expression. Moreover, TSG promoted Sirtuin 1 (SIRT1) expression. When SIRT1 was inhibited by EX527 or SIRT1 siRNA, the effect of TSG is diminished according to the increased proportion of cells in the G1 phase, cell apoptosis, p21, and PAI-1 expression. Overall, our study established TSG as an anti-senescence compound that exerts its protective action by regulating SIRT1 expression.

## Introduction 

Many studies have shown that endothelial cell senescence and dysfunction are the key factors leading to cardiovascular injury (Lin et al., 2017). Oxidative stress is a vital cause of endothelial dysfunction and senescence. Many age/longevity-related regulators have been described, such as Sirtuins (SIRTs), FOXO transcription factor, and mitogen-activated protein kinase (Harvey et al., 2015). SIRTs are a class of proteins that possess NAD^+^-dependent deacetylase activity (Kida and Goligorsky, 2016). Among the SIRT family, Sirtuin 1 (SIRT1) is currently thought to exert vascular protection and considered an anti-senescence molecule and is implicated in diverse cellular processes, including differentiation, senescence, apoptosis, metabolism, oxidative stress response, and inflammation (Michan and Sinclair, 2007; Yamakuchi and Lowenstein, 2009; Morris et al., 2011). And overexpression of SIRT1 in endothelial cells may prevent cellular senescence.However, the accurate mechanism of vascular aging is still largely unknown, and effective treatments are still lacking.

2,3,5,4’-tetrahydroxy-stilbene-2-O-β-d-glucoside (TSG) is one of *Polygonum multiflorum* main active ingredients (Jiang et al., 2017), which has been shown to possess many biological properties such as its anti-inflammatory (Chin et al., 2016), antioxidant (Ling et al., 2016), anti-aging, and anti-atherosclerosis properties (Lin et al., 2018). Wang et al. (2009) proposed that TSG enhances the expression of SIRT1 and protects against cerebral ischemia/reperfusion injury. These findings led us to investigate the potential role of TSG in HUVEC senescence and the mechanism by which it exerts its effect on vascular prevention.

This study aimed to demonstrate whether TSG can relieve the HUVEC senescence and investigate how SIRT1 functions in the process of HUVEC senescence when TSG is present.

## Materials and Methods

### Materials

The HUVEC were purchased from the Institute of Biology, Chinese Academy of Sciences. The 2,3,5,4’-Tetrahydroxystilbene-2-O-β-D-Glucoside was purchased from Sigma company, American (lot: M48818078). And TSG was dissolved in sterile water.

### Cell Culture and Treatment

HUVEC were cultured in RMPI 1640 medium (Gino, Hang Zhou, China) containing 10% heat-inactivated fetal bovine serum (FBS, Sigma, American) and 1% penicillin-streptomycin at 37°C in 5% CO_2_. HUVECs were pretreated with various concentrations of TSG (20 and 40 μg/ml) 24 h. Then HUVECs were treated with H_2_O_2_ (0.2 mM) 2 h and cultured in normal medium for 24 h later. As for EX527, the specific inhibitor of SIRT1, were added 40 μM for 24 h before pretreating with TSG.

### MTS Assay

The MTS assay was used to assess cell viability. Before each experiment, HUVEC (5,000 cells/well) were seeded in 96-well microtiter plates and treated. Subsequently, 20 μl MTS solution was added to each well, and the plates were incubated for 2 h at 37°C. The absorbance was measured at 490 nm and used to calculate the relative ratio of cell viability.

### Senescence-Associated β-Galactosidase Activity

Senescence was assessed by β-galactosidase staining. Cells were washed in PBS and then fixed with fixation fluid for 15 min at room temperature. After washing three times, cells were incubated at 37°C overnight with dyeing liquor. At the end of the incubation, five random fields were counted per dish to assess the percentage of senescence-associated beta-galactosidase (SA-β-gal) positive cells.

### Cell Cycle Analysis

The cell cycle was detected by flow cytometry-based on propidium iodide (PI) staining. Cells were collected and fixed overnight with 70% alcohol at 4°C. The cells were centrifuged to remove the alcohol and washed twice with cold PBS. Next, the PI stain was added, and the cells were protected from light for 30 min at 4°C. Cell cycles were analyzed, and the percentage of cells in the G1 phase was recorded.

### Annexin V-FITC/PI Dual Staining

Cell apoptosis analysis was performed using the Annexin V-FITC Apoptosis Detection Kit (Beyotime). Briefly, after experimental treatments, the cells were washed twice with cold PBS and resuspended in 100 μl binding buffer, followed by incubation with 5 μl Annexin V-FITC and 10 μl PI at room temperature for 15 min. A total of 10,000 cells were collected and analyzed by flow cytometry (BD Biosciences, Franklin Lakes, NJ, USA) equipped with a Cell Quest software (BD Biosciences).

### Immunofluorescence

HUVEC were fixed for 30 min at room temperature in 4% paraformaldehyde. Next, HUVEC were treated with PBS containing 0.5% Triton X-100 for 10 min and were then blocked with 5% BSA for 1 h. Cells were subsequently incubated with SIRT1 antibody (1:1,000 in PBS with 0.1% Triton X-100 and 5% BSA) overnight at 4°C and then incubated with anti-rabbit secondary antibody at room temperature for 2 h. Chromosomes were stained with DAPI for 5 min. The HUVEC were mounted on glass slides and examined with a laser scanning confocal microscope (Zeiss LSM 880).

### Western Blot

Briefly, proteins were extracted with radio immunoprecipitation assay (RIPA) solution, and the concentration was measured with a bicinchoninic acid (BCA) kit (Beyotime, China). Protein samples were separated by SDS-PAGE using a 10% polyacrylamide gel. Then, membranes were exposed to anti-SIRT1 (1:1,000 dilution), anti-p21 (1:2,000 dilution), anti-PAI-1 (1:1,000 dilution), and anti-GAPDH (1:2,000 dilution) overnight at 4°C. The membranes were washed (three times, 10 min each) in Tris-buffered saline (TBS) containing 0.1% Tween-20 (TBST) and then incubated with the corresponding secondary antibody.

### Isolation of RNA and Real-Time Fluorescence Quantitative PCR

Total RNA was extracted with TRIzol reagent. The complementary DNA was synthesized using Prime Script TM RT Master Mix (Takara, Dalian, China) for reverse transcription PCR with conditions of 37°C for 15 min, 85°C for 5 s, and storage at 4°C. The Real-time fluorescence quantitative PCR was performed in duplicate using SYBR^®^Prime Ex Taq TM II (Tli RNaseH plus) (Takara, Dalian, China) at 95°C for 2 min, and then 56°C for 30 s, 72°C for 30 s for 40 cycles. The primer sequences used in this study were as follows: SIRT1 F: ACTTCAGGTCAAGGGATG, and R: CACTGCACAGGCACATAC.

### siRNA Transfection

Cells in the exponential phase of growth were plated in six-well plates at 2 × 10^5^ cells/plate and cultured for 24 h. Then, the cells were transfected with SIRT1 siRNA (30 pmol) 12 h using Lipofectamine RNAiMAX transfection reagent (Invitrogen) according to the manufacturer’s protocols.

### Statistical Analysis

All experiments were performed independently in triplicate. The results are presented as mean ± SD calculated by GraphPad Prism. For multiple comparisons, one-way ANOVA followed by Tukey *post hoc* test was performed. The results were considered to be statistically significant when *p <*0.05.

## Results

### TSG Prevented H_2_O_2_-Induced Injury in HUVEC

**Figure 1:**
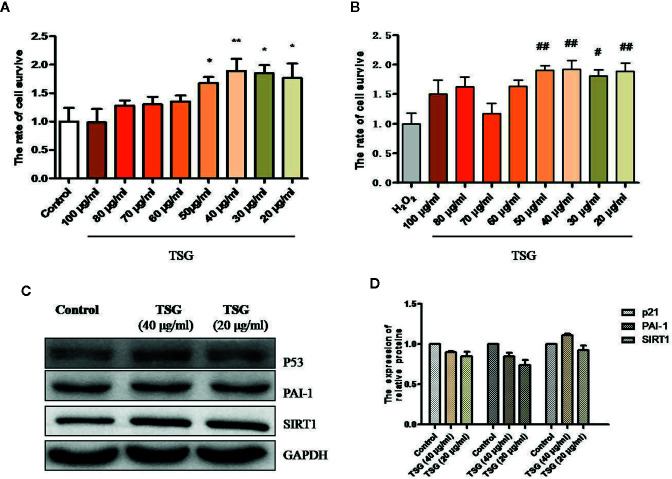
TSG rescued H_2_O_2_-induced injury in HUVEC. **(A)** MTS assay was to detect that wheteher TSG at concentrations could damage the HUVEC viability or not. **(B)** MTS assay was to detect that wheteher TSG could promote the HUVEC viability or not under H_2_O_2_ condition. **(C, D)** Representative images of WB analysis and the semi-quantification of p21, PAI-1, and SIRT1 in normal HUVEC pretreated with TSG. The values are expressed as the mean ± SD (n = 3). *p <0.05 and **p < 0.01 vs. control, ^#^ *p < *0.05 and ^##^ *p <* 0.01 vs. H_2_O_2_.

MTS assay was performed to determine the effective dose of TSG on HUVEC for 24 h exposure. TSG at concentrations did not damage the HUVEC viability (**Figure 1A**). Pretreatment of HUVEC with TSG (20–50 μg/ml) attenuated injury in 0.2 mM H_2_O_2_-treated HUVEC (**Figure 1B**). TSG (20 and 40 μg/ml) had no effect on the expression of p21, plasminogen activator inhibitor-1 (PAI-1), and SIRT1 in normal HUVEC (**Figures 1C, D**). So, TSG at two concentrations (20 and 40 μg/ml) were choosed to experience further study.

### TSG Can Prevent HUVEC Senescence

**Figure 2:**
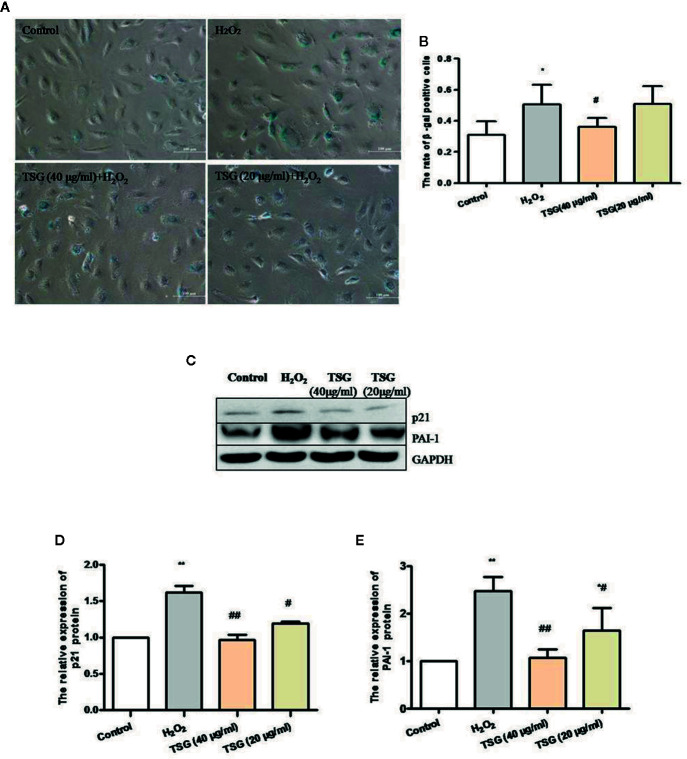
TSG can prevent HUVEC aging. **(A)** Representative images of senescence-associated β-gal staining in HUVEC pretreated with TSG under H_2_O_2_ induction. **(B)** Quantification of SA-β-gal positive cell number from three biological replicates. For each replicate, at least 5 random microscopic fields were counted. **(C–E)** Representative images of WB analysis and the semi-quantification of PAI-1 and p21 in HUVEC pretreated with TSG under H_2_O_2_ induction. The values are expressed as the mean ± SD (n = 3). *p <0.05 and **p < 0.01 vs. control, ^#^ *p < *0.05 and ^##^ *p < *0.01 vs. H_2_O_2_.

In order to identify the effect of TSG on H_2_O_2_-induced senescence in cultured HUVEC, we evaluated SA-β-gal activity, PAI-1 expression, and p21 expression, which are characteristic indicators of cellular senescence. We found that exposure of HUVEC to 0.2 mM H_2_O_2_ markedly increased SA-β-gal activity and the expression of PAI-1 and p21, whereas pretreatment with TSG significantly suppressed SA-β-gal activity and the expression of PAI-1 and p21 (**Figures 2A–E**).

### TSG Mitigated H_2_O_2_-Induced Senescence and Apoptosis

**Figure 3:**
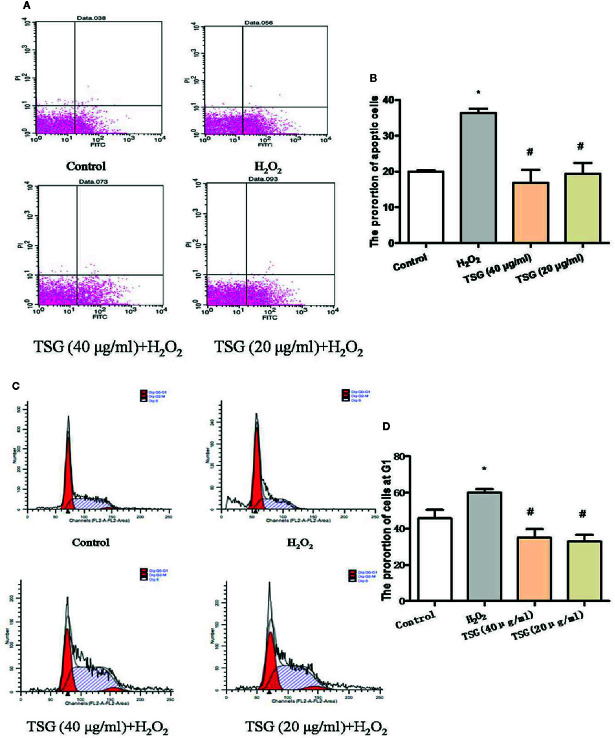
TSG mitigated H_2_O_2_-induced senescence and apoptosis. **(A–D)** Representative images of flow cytometry analysis and quantification of cell cycle proportion or apoptosis in HUVEC pretreated with TSG under H_2_O_2_ induction. The values are expressed as the mean ± SD (n = 3). *p <0.05 vs. control, ^#^ *p < *0.05 vs. H_2_O_2_.

The growth cycle of senescent cells will stagnate in the G1 phase. And the ultimate fate of senescent cells is cell death, which is a kind of apoptosis. As shown in **Figures 3A–D**, the treatment of HUVEC with 0.2 mM H_2_O_2_ significantly increased the proportion of HUVEC in the G1 phase and the apoptosis rate. Preincubation with TSG significantly attenuated H_2_O_2_-induced senescence and apoptosis.

### TSG Promoted SIRT1 Expression in H_2_O_2_-Treated HUVEC

**Figure 4:**
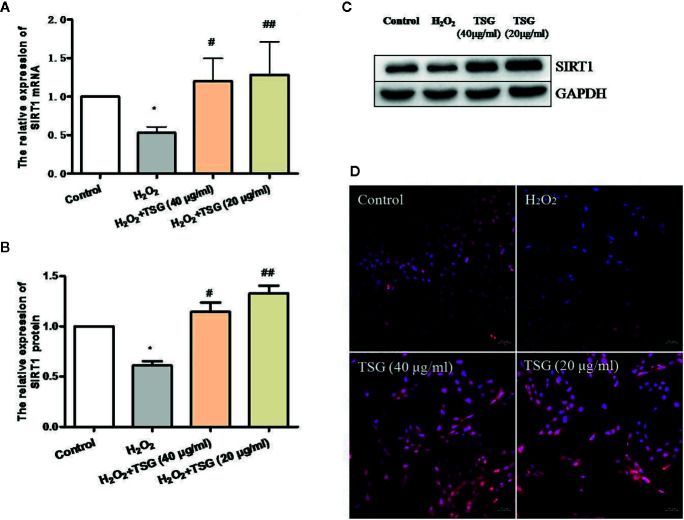
TSG promoted SIRT1 expression in H_2_O_2_-induced HUVEC. **(A)** Real-time fluorescence quantitative PCR was used to detect the expression of SIRT1 mRNA. **(B, C)** Representative images of WB analysis and the semi-quantification of SIRT1. **(D)** Representative images of staining with DAPI (blue) and SIRT1-specific fluorometric probe (red) acquired using a laser scanning microscope. Values are expressed as mean ± SD (n = 3); *p <0.05 vs. control, ^#^p < 0.05 and ^##^p < 0.01 vs. H_2_O_2_.

SIRT1 was usually associated with longevity. So, we speculated that it may become a target of anti-senescence drugs. As shown in **Figures 4A–D**, H_2_O_2_ treatment decreased the expression of SIRT1, while TSG (40 μg/ml) significantly increased SIRT1 mRNA and protein expression. In order to more directly observe the effect of TSG on the expression of SIRT1 in HUVEC, immunofluorescence experiments were performed showing that TSG can significantly increase the fluorescence intensity associated with SIRT1 in senescent HUVEC, suggesting that TSG may block H_2_O_2_-induced HUVEC senescence *via* SIRT1.

### TSG Reduced the Expression of H_2_O_2_-Induced HUVEC Senescence-Related Proteins *via* Regulating SIRT1

**Figure 5:**
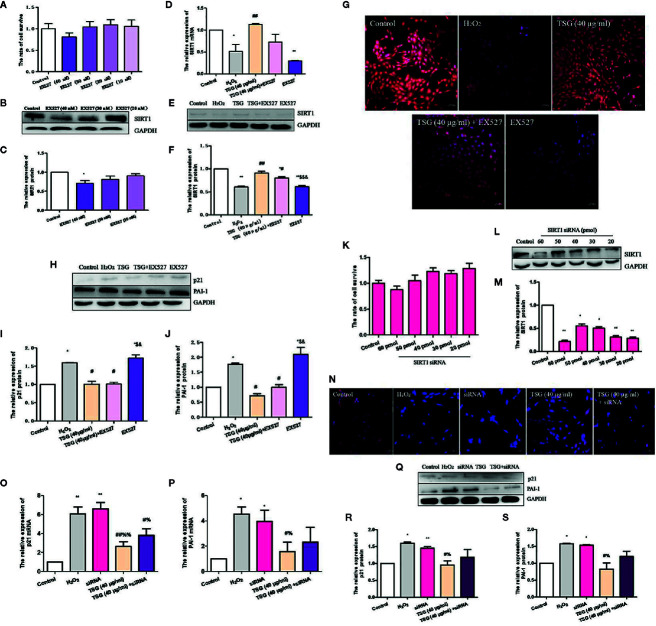
TSG reduced the expression of H_2_O_2_-induced HUVEC senescence-related proteins *via* regulating SIRT1. **(A)** MTS assay was to detect that wheteher EX527 at concentrations could damage the HUVEC viability or not. **(B, C)** Representative images of WB analysis and the semi-quantification of SIRT1 at different concentrations of EX527 in normal HUVEC. **(D)** Real-time fluorescence quantitative PCR was used to detect the expression of SIRT1 mRNA. **(E, F)** Representative images of WB analysis and the semi-quantification of SIRT1 in HUVEC pretreated with EX527 and TSG under H_2_O_2_ induction. **(G)** Representative images of staining with DAPI (blue) and SIRT1-specific fluorometric probe (red) acquired using a laser scanning microscope Representative images of WB analysis and the semi-quantification of SIRT1. **(H–J)** Representative images of WB analysis and the semi-quantification of PAI-1 and p21 in HUVEC pretreated with EX527 and TSG under H_2_O_2_ induction. **(K)** MTS assay was to detect that wheteher SIRT1 siRNA at concentrations could damage the HUVEC viability or not. **(L, M)** Representative images of WB analysis and the semi-quantification of SIRT1 at different concentrations of SIRT1 siRNA in normal HUVEC. **(N)** Representative images of staining with DAPI (blue) and SIRT1-specific fluorometric probe (red) acquired using a laser scanning microscope Representative images of WB analysis and the semi-quantification of SIRT1. **(O, P)** Real-time fluorescence quantitative PCR was used to detect the expression of PAI-1 and p21 mRNA. **(Q–S)** Representative images of WB analysis and the semi-quantification of PAI-1 and p21 in HUVEC pretreated with SIRT1 siRNA and TSG under H_2_O_2_ induction. The values are expressed as the mean ± SD (n = 3). *p <0.05 and **p <0.01 vs. control, ^#^ *p <*0.05 and ^##^ *p <*0.01 vs. H_2_O_2_, ^$^ *p <*0.05 and ^$$^ *p <*0.01 vs. TSG group, ^&^ *p <*0.05 vs. TSG + EX527 group, ^%^ *p <*0.05 and ^%%^ *p <*0.01 vs. siRNA group.

In order to determine whether TSG mitigates H_2_O_2_-induced HUVEC senescence by promoting the expression of SIRT1, we used EX527, a specific inhibitor of SIRT1. EX527 did not induce significant cytotoxicity in normal HUVEC (**Figure 5A**). Treatment with 40 nM EX527 significantly decreased SIRT1 expression (**Figures 5B, C**). Moreover, when SIRT1 was inhibited by EX527, TSG cannot change the SIRT1 expression significantly in H_2_O_2_-induced HUVEC. (**Figures 5D–G**). Additionally, we analyzed the expression of senescence-associated proteins. When SIRT1 expression was inhibited, the expression of the PAI-1 and p21 protein had no significant difference between the H_2_O_2_ group and EX527 group. These determined that the expression of SIRT1 was associated with HUVEC senescence. And the expression of the PAI-1 and p21 in the TSG+EX527 group were higher than those in the TSG group (**Figures 5H–J**). Therefore, we can partly believe that TSG alleviated H_2_O_2_-induced HUVEC senescence *via* regulating SIRT1.

Similarly, we also transfected SIRT1 siRNA to silence SIRT1 expression. SIRT1 siRNA at concentrations did not damage the HUVEC viability (**Figure 5K**). We found that 30 pmol SIRT1 siRNA can silence SIRT1 successfully (**Figures 5L, M**). When HUVEC was infected with SIRT1 siRNA, immunofluorescence experiments were performed. The results showed that TSG had little effect on increase the fluorescence intensity associated with SIRT1 in senescent HUVEC (**Figure 5N**). And when the expression of SIRT1 was silenced, the degree of HUVEC senescence increased and the anti-senescence effect of TSG was weakened, which indicated by the expressions of PAI-1 and p21 mRNA and protein (**Figures 5O–S**). Thence, we have better reasons to believe that TSG mitigated HUVEC senescence through SIRT1.

### TSG Affected H_2_O_2_-Induced HUVEC Cell Cycle and Apoptosis by Regulating SIRT1

**Figure 6:**
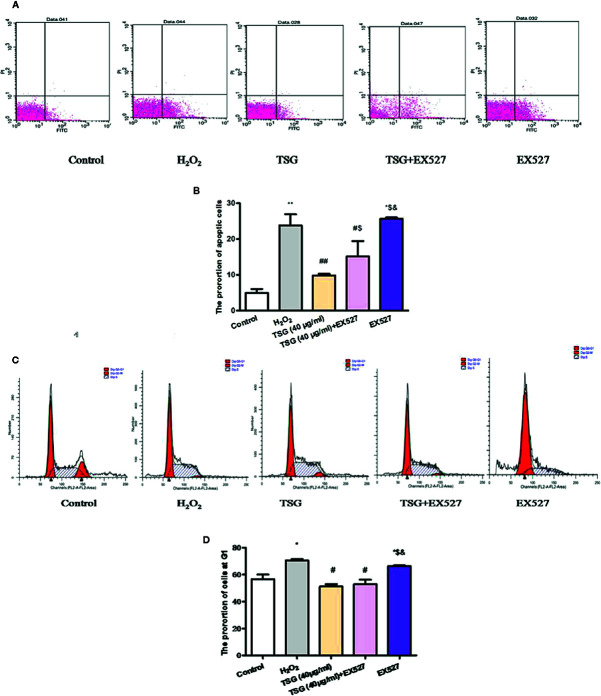
TSG affected H_2_O_2_-induced HUVEC cell cycle and apoptosis by regulating SIRT1. **(A–D)** Representative images of flow cytometry analysis and quantification of cell cycle proportion or apoptosis in HUVEC pretreated with TSG under H_2_O_2_ induction when the expression of SIRT1 was inhibited. The values are expressed as the mean ± SD (n = 3). *p <0.05 and **p <0.01 vs. control, ^#^ *p <*0.05 and ^##^ *p <*0.01 vs. H_2_O_2_, ^$^ *p <*0.05 and ^$$^ *p <*0.01 vs. TSG group, ^&^p<0.05 vs. TSG + EX527 group.

In order to know whether TSG could affect H_2_O_2_-induced HUVEC cell cycle and apoptosis by regulating SIRT1 or not, we examined the proportion of cells in the G1 phase and the rate of apoptosis under the SIRT1 inhibited condition. When SIRT1 expression was inhibited, the proportion of HUVEC in the G1 phase and the rate of apoptosis had no significant difference between the H_2_O_2_ group and EX527 group. What is more, the results showed that the proportion of HUVEC in the G1 phase and the rate of apoptosis in the TSG+EX527 group was significantly higher than in the group treated with TSG alone (**Figures 6A–D**). Therefore, inhibition of SIRT1 expression antagonized the prevention of H_2_O_2_-induced senescence and apoptosis by TSG in HUVEC, which was another supporting proof for TSG eased HUVEC senescence through SIRT1.

## Discussion

Cell senescence is a phenomenon of cell growth arrest induced by cells subjected to some stressors such as oxidative stress or DNA damage (Regulski, 2017). The senescence of endothelial cells weakens the basic functions of cells, and the imbalance of cell functions promotes the development and progress of the cardiovascular disease. Therefore, anti-vascular endothelial cell oxidative senescence plays an important role in the prevention of cardiovascular disease (Regina et al., 2016). Our results showed that TSG can alleviate the HUVEC stress-induced premature senescence.

TSG, a resveratrol analog with glucoside, has been shown to prevent vascular endothelial dysfunction (Zhang et al., 2019). We evaluated molecular markers of senescence, including SA-β-gal (Piechota et al., 2016), PAI-1 (Baker et al., 2011), and p21 (Inoue et al., 2009), confirming that TSG has a protective effect on H_2_O_2_-induced HUVEC. And the results showed that TSG can significantly reduce HUVEC dysfunction by blocking H_2_O_2_-induced oxidative stress and reducing the expression of SA-β-gal, PAI-1, and p21.

Endothelial cell senescence will show obvious changes in characteristics, leading to impaired vascular function and neoangiogenesis (Regina et al., 2016). Senescent cells are usually bulky and have high β-galactosidase activity at pH 6.0 (Vaughan et al., 2017). In the detection of cell SA-β-gal activity, we found that TSG seems to reduce the stress response of cells by inhibiting its enzyme activity. PAI-1 is a member of the serine protease inhibitor family. As a major marker of cellular senescence, its overexpression in the body guides the occurrence and progression of a variety of human diseases and promotes various incidences of senescence. Related studies have shown that a significant positive correlation has been established between elevated levels of PAI-1 and senescence-related β-galactosidase positive cells, confirming that PAI-1 is an endothelial cell senescent excellent marker (Xu et al., 2000). p21 is a pleiotropic inhibitor of the cyclin-dependent kinase complex located downstream of p53, which mediates the progression of the cell cycle (Dotto, 2000). And p21 has an inhibitory effect on cells, it can mediate cell cycle arrest, induce cell growth arrest and senescence when cells are damaged (Shtutman et al., 2017). The experimental results were in line with expectations, TSG reduced the blue staining rate of SA-β-gal cells and down-regulated the expression of PAI-1 and p21. Combined with subsequent apoptosis and cycle analysis, TSG did significantly alleviate the process of endothelial cell injury or senescence-induced by H_2_O_2_. Besides, we found that TSG can rescue the expression of SIRT1 in H_2_O_2_-induced HUVEC.

SIRT1 is a negative regulator of cell senescence (Yao et al., 2012) and is highly expressed during angiogenesis in endothelial cells. Disruption of SIRT1 abrogates vascular endothelial homeostasis and remodeling (He et al., 2017). High SIRT1 levels have been shown to inhibit oxidative stress and DNA damage (Zhao et al., 2010). Besides, a study reported that aging-related SIRT1 loss in VSMCs causes impaired stress responses and increased senescence, confirming the previous point (Thompson et al., 2014). In our results, SIRT1 had a beneficial effect on the function of TSG in H_2_O_2_-induced HUVEC senescence. Cell cycle and apoptosis detection using cell flow cytometry, and results showed that SIRT1 reduced the state of cell growth arrest in the G1 phase and significantly reduced the cell’s apoptosis rate. It can be speculated that TSG treatment stimulated the expression of SIRT1, thereby reducing the senescence and even apoptosis of HUVEC. But under conditions of low SIRT1 expression, that is, when SIRT1 was inhibited by EX527 or SIRT1 siRNA, the expression level of PAI-1 and p21 (cell senescence markers) proteins increased, and TSG had a little protective effect on HUVEC senescence. The expression level of PAI-1 and p21 proteins in SIRT1 siRNA group were lower than those in H_2_O_2_ group, which was consistented with the results in the literature (Yang et al., 2013; Guo et al., 2019). As for the diffenence between the expression of p21 mRNA and protein, there are further studies in needed. Therefore, TSG had a positive effect on the senescence of endothelial cells by regulating the expression of SIRT1. According to previous reports, in the initial study of SIRT1 activation by resveratrol, there has been increasing interest in developing more effective SIRT1 activators to treat related diseases (Lagouge, 2016; Milne et al., 2017). Combined with our results, TSG reduced HUVEC oxidative senescence by regulating SIRT1 expression. So, whether TSG can develop a more effective SIRT1 activator to delay senescence or treat related diseases is worth further research.

## Conclusions

In conclusion, oxidative stress has been suggested to play a role in cellular senescence and in human aging in general and is an inducement for the development of various cardiovascular diseases. Therefore, anti-oxidation treatment for aging or various cardiovascular diseases is an important research direction in the future. In our study, H_2_O_2_ can induce HUVEC senescence successfully. And TSG not only confirmed its protective effect on HUVEC senescence but also found that SIRT1 is its target for reducing HUVEC senescence.

## Data Availability Statement

The datasets generated for this study are available on request to the corresponding author.

## Author Contributions

BJ and HW conceived and designed the study. YG, XY, and SC performed the experiments and analyzed the data. YG wrote the paper. YG and WF reviewed and edited the manuscript. All authors contributed to the article and approved the submitted version.

## Funding

This work was supported by the Natural Science Foundation of Zhejiang Province, China (LY17H280007); and the National Natural Science Foundation of China (No.81630105).

## Conflict of Interest

The authors declare that the research was conducted in the absence of any commercial or financial relationships that could be construed as a potential conflict of interest.
